# Tuning the Electronic Properties of Mesocrystals

**DOI:** 10.1002/smsc.202200014

**Published:** 2022-06-28

**Authors:** Christian Jenewein, Stefan M. Schupp, Bing Ni, Lukas Schmidt-Mende, Helmut Cölfen

**Affiliations:** ^1^ Department of Chemistry University of Konstanz Universitätsstraße 10 78462 Konstanz Germany; ^2^ Department of Physics University of Konstanz Universitätsstraße 10 78462 Konstanz Germany

**Keywords:** colloidal crystals, electrical conductivity, mesocrystals, nanocubes, nanoprobing platinum, self-assembly

## Abstract

Colloidal crystals are arguably one of the most promising candidates when it comes to the fabrication of nanostructured metamaterials. Especially mesocrystals show exciting new properties that emerge from their inherent directional oriented assembly. With this work, the electrical conductivity of well‐defined micrometer‐sized platinum nanocube‐based mesocrystals is demonstrated and tuned through the variation of different capping agents. Herein, a method is presented to reproducibly quantify the intrinsic resistance of individual mesocrystals through electrical nanoprobing and focused ion beam deposition contacting. A thermally activated tunneling mechanism is identified as the main effect for electron propagation. In addition, the mesocrystals are altered through organically linking and mineral bridging the individual nanoparticles. This results in an increase in mesocrystal rigidity and, more importantly, conductivity by seven orders of magnitude while retaining shape, structure, and composition. In addition, these observations are transferred onto multicomponent superstructures in the form of binary mesocrystals. There, it is demonstrated that the electrical properties could be tuned through the ratio of nanoparticles incorporated into a mesocrystalline host system while simultaneously maintaining potential catalytic or superparamagnetic features of the guest particles.

## Graphical abstract

Nanostructured metamaterials in the form of monophase or even binary mesocrystals are subject to fundamental research in recent literature. Herein, a more application‐oriented property—the electrical conductivity—is investigated in great detail and reveals the vast potential that such multicomponent superstructures can inhere.

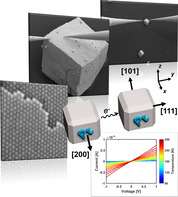

## 1 Introduction

The discovery of the spontaneous self‐assembling behavior of nanocrystals into highly ordered superstructures has given rise to a new generation of nanostructured materials.^[^ ^1^ ^]^ This type of condensed matter is often referred to as “supercrystals” due to their astounding new characteristics and unique functionalities that arise from the collective properties of highly ordered nanocrystals.^[^ ^2^ ^]^ Extensive research has shown how spherical nanoparticles commonly self‐organize into close‐packed face‐centered cubic (fcc) or hexagonal close‐packed (hcp) superlattices as a result of van der Waals interactions.^[^ ^3^ ^]^ These so‐called colloidal crystals gained a lot of attention in recent years as the understanding of their physical properties is a crucial part of the formation of metamaterials.^1^, ^4^ While isotropic nanocrystals are highly efficient in forming colloidal crystals with a large space‐filling fraction, they lack directional properties and therefore are inherently limited in their properties.^3^, ^5^ The focus on anisotropic nanoparticles and their ability to form crystallographically oriented arrangements within colloidal superstructures gave rise to a new type of colloidal crystals named mesocrystals.^4^, ^6^ In 2005, a clear and systematical description was proposed in which mesocrystals form from nonspherical crystalline building units as oriented superstructures with common outer faces.^[^ ^7^ ^]^

Due to the unique process through which these mesocrystals emerge, they further attracted a lot of attention when it comes to the investigation of the underlying nonclassical crystallization processes.^4^, ^8^ Advanced crystallographic techniques revealed that there is a need for a more distinct description of mesocrystals, which is based on the International Union of Crystallography (IUCr) definition of crystals as “a nanostructured material with a defined long‐range order on the atomic scale (in at least one direction), which can be inferred from the existence of an essentially sharp wide‐angle diffraction pattern (with sharp Bragg peaks) together with clear evidence that the material consists of individual nanoparticle building units.”^[4c]^ In addition to the crystallographic complexity, such mesocrystals inhere, recent research shows that the evolving properties of tailored mesocrystals that arise when ordering anisotropic nanoparticles can open a whole new range of yet unknown features such as superparamagnetism in large particle assemblies.^[^ ^9^ ^]^ Especially their common appearance in high‐performing biological matter such as nacre further displays the significance of their structure–property relationships.^[^ ^10^ ^]^ However, the formation of anisotropic nanoparticles into mesocrystalline superstructures on the micrometer scale remains a major challenge up until this day. A complex interplay of multiple driving forces among nanocrystals, stabilizing agent, and solvent molecules requires a precise tuning of the experimental conditions to develop both orientational and translational orderings.^8^, ^11^ From all the anisotropic nanoparticles, the nanocube is the simplest and most commonly synthesized of the five platonic bodies for a wide range of materials.^9^, ^12^ The structural anisotropy with varying crystallographic properties between corner, edge, and flat facet allows the exploration of shape‐dependent structural and physical properties such as the electronic coupling of neighboring metallic nanoparticles as a result of the extended facet‐to‐facet contact area. While the structure and arrangement of particles provide a large contribution toward the properties of such metamaterials, the actual composition, of course, remains the deciding factor for its properties. In terms of physical and electrochemical properties, elemental platinum has proven to be the most versatile and promising candidate in the development of advanced functional materials that inhere enhanced and/or novel properties for industrial applications.^[^ ^13^ ^]^ Furthermore, platinum nanoparticles have repeatedly demonstrated their emerging properties and enhanced activity in electrocatalysis, as well as their ability to self‐assemble into superstructures.^[^ ^14^ ^]^ Due to the importance of conductivity within these materials, we seek to study its effects and underlying mechanism within the scope of this work. We, therefore, investigate colloidal crystals in form of micrometer‐sized platinum nanocube (PtNC)‐based mesocrystals by unraveling their electrical properties and how they can be tuned through capping agents or heat treatment.^[^ ^15^ ^]^ With our recent discoveries of artificially tailored mesocrystals, we compare our results to the conductivity of magnetite nanocube‐based mesocrystals and further explore how a combination of platinum and magnetite nanoparticles into binary mesocrystals can affect the conductivity of such assemblies.^14^, ^16^

## 2 Discussion

**Figure 1:**
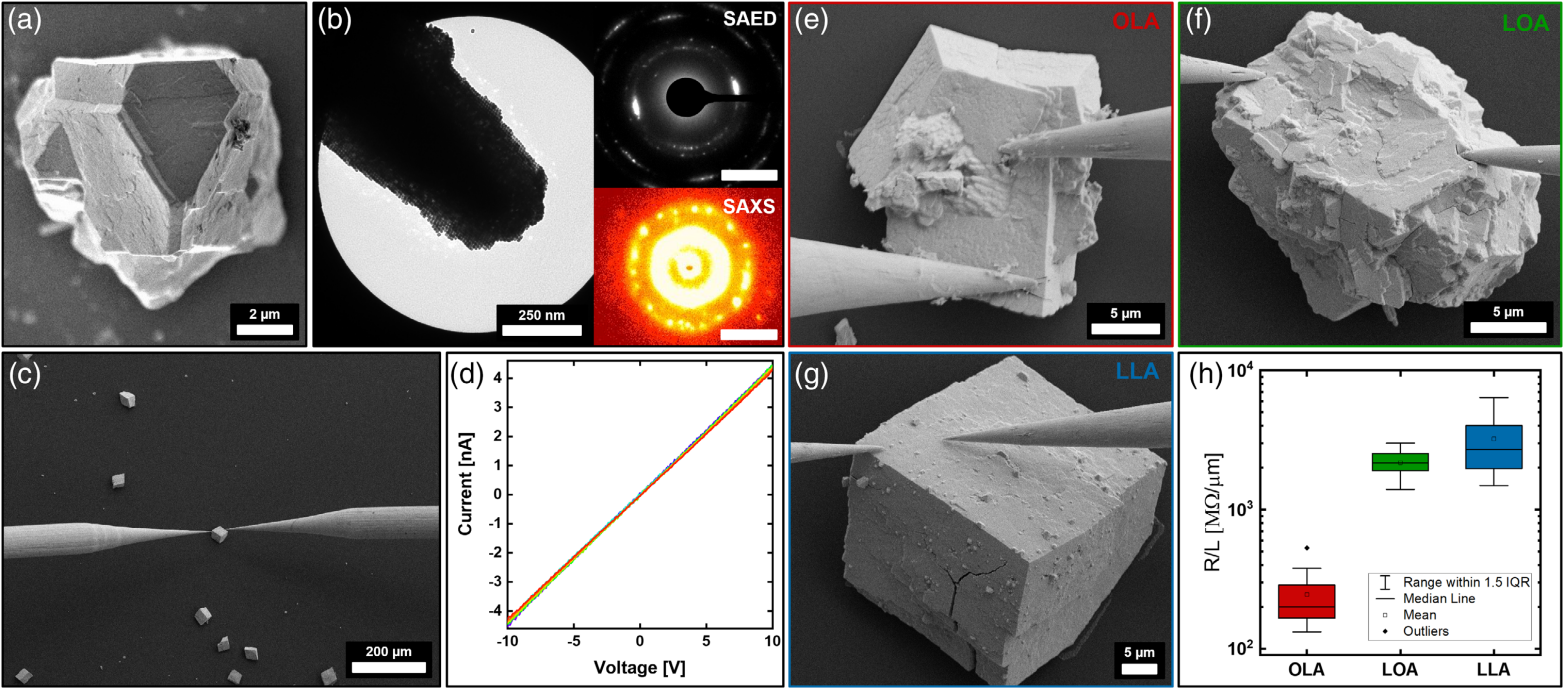
a) The scanning electron microscopy (SEM) image of an oleic acid (OLA)‐stabilized platinum nanocube (PtNC) mesocrystal identified as such by a selected area electron diffraction (SAED) performed on a fractured crystal b) and the corresponding small‐angle X‐Ray scattering (SAXS) analysis of multiple mesocrystals (b inset). c) A field‐emission SEM (FE‐SEM) image of two 200 nm tungsten nanoprobing tips contacting a single mesocrystal and d) the corresponding current–voltage (*I–V*) curves over five consecutive measurements. FE‐SEM images (e, OLA), (f, linoleic acid [LOA]), and (g, linolenic acid [LLA],) show a closeup example of all three different mesocrystal types during conductivity measurements. The corresponding results of the ratio between measured resistance *R* and respective nanoprobe tips separation *L* is illustrated in h) in the form of a box plot diagram. Scale bar in (b, inset) is 5 nm^−1^ for SAED and 0.1 q for SAXS.

All PtNCs used in this work were obtained by utilizing a slightly modified heating‐up synthesis originally reported by Zhang et al.^[14c]^ In total, three different PtNC batches stabilized by either oleic acid (OLA, (9Z)‐Octadec‐9‐enoic acid), linoleic acid (LOA, (9Z,12Z)‐Octadeca‐9,12‐dienoic acid), or linolenic acid (LLA, (9Z,12Z,15Z)‐Octadeca‐9,12,15‐trienoic acid) were produced and self‐assembled analogous to a method which has been established and investigated in detail in prior work.^[14a]^ In preparation for the experiments, the PtNC dispersions were first purified by recrystallization from hexane according to a previously reported method to narrow its particle size distribution and remove particles with a significantly increased aspect ratio.^14^, ^17^ The resulting average particle sizes of the PtNC dispersions have been determined to be 10.7 ∓ 1.0 nm for OLA, 10.8 ∓ 2.1 nm for LOA, and 12.3 ∓ 2.5 nm in the case of LLA with an aspect ratio slightly above 1.3 in all three batches (Supporting Information 1). Through a standard gas‐phase diffusion technique, PtNCs were self‐assembled from hexane into highly ordered mesocrystals on a silicon dioxide–coated silicon wafer substrate using ethanol as the primary dispersion agent. Prior to crystallization, a marked grid was engraved onto the substrate through photolithography to exactly locate individual mesocrystals between the following treatments and measurements (Supporting Information 2, Figure S2, Supporting Information). The so‐obtained mesocrystals have been identified as such via scanning electron microscopy (SEM), small‐angle X‐Ray scattering (SAXS), and selected area electron diffraction (SAED) techniques as displayed in **Figure** **1**a,b. Conductivity measurements were then performed on the mesocrystals by contacting two tungsten nanoprobing tips with a diameter of 200 nm within the vacuum chamber of a field‐emission SEM (FE‐SEM) as shown in Figure 1c and S3, Supporting Information. The physical structure of the mesocrystals appears to be of a soft, buttery texture, which allowed for a sufficient contact area of the measuring tip when applying a small force. In a crosscheck experiment, we utilized 2000 nm sized tungsten nanoprobing tips and compared their results to previous measurements to exclude the emerging contact resistance as a main factor (Supporting Information 2, Figure S4a, Supporting Information). Five consecutive voltage sweeps (forward and backward) showed Ohmic and reproducible current–voltage (*I–V*) curves for the different mesocrystals (Figure 1d). For comparison, the ratio of measured zero‐bias resistances *R* and respective distances *L* between both nanoprobe tips was calculated assuming a linear increase in resistance for larger distances. To rule out that the conductivity is caused by a surface‐altering effect during the measurement, the mesocrystals have been placed on a substrate with prefabricated Au electrodes and measured across opposing surfaces of the crystal (Supporting Information 2, Figure S5, Supporting Information). The obtained values for the conductivity are within the range of our previous measurements. This implies that the performed two‐point measurements at mesocrystal surfaces also reflect the electron‐conducting mechanism within its bulk structure. To account for variations in crystal growth, we measured 10 mesocrystals each and averaged their results within a box plot graph as illustrated in Figure 1e–h. Mesocrystals using LOA and LLA as capping agents show a high resistance with mean ratios of 2169 and 3220 MΩ μm^−1^, respectively. Therefore, the LOA and LLA samples display a minor noise within their measuring curves due to their resistances being close to the detection limit of the instrument (Supporting Information 2, Figure S3, Supporting Information). In contrast, the OLA samples exhibit an order of magnitude higher conductivity with a resistance of 245 MΩ μm^−1^ (Figure 1h). Furthermore, we were not able to record any significant variations when measuring the resistance parallel or diagonal to the mesocrystals outer facets.

**Figure 2:**
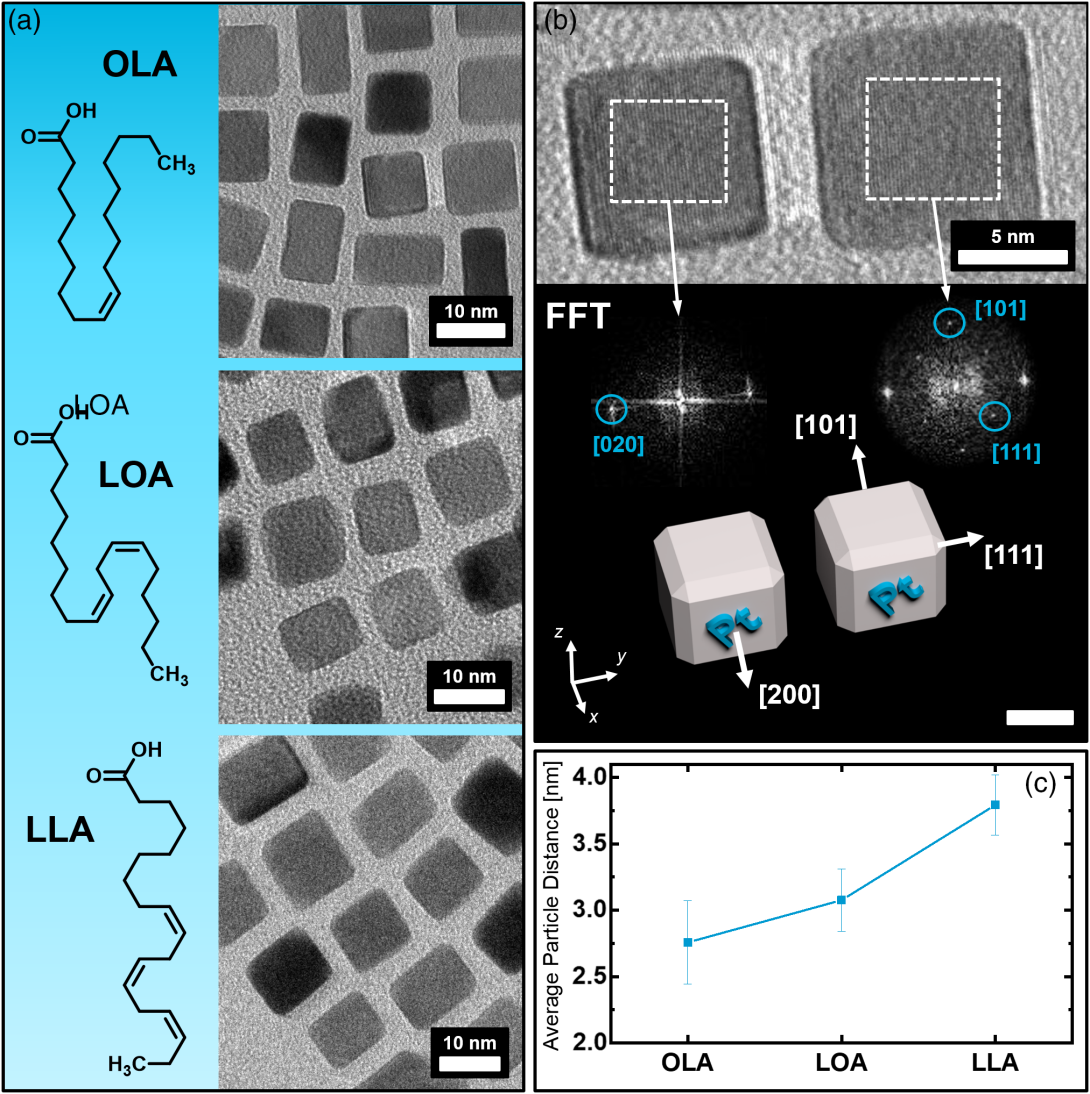
High‐resolution transmission electron microscope (HR‐TEM) images and the molecular structures of 2D self‐assembled PtNCs stabilized by a) the three different fatty acids OLA, LOA, and LLA. b) Two OLA‐stabilized PtNCs within a particle arrangement at a magnification of 200 kX allowing for a precise determination of the particle distance. Fast Fourier transformation (FFT) analysis of the lattice planes gives platinum specific signals for a [100] zone axis revealing the exact crystallographic orientation as illustrated by the modeled nanocubes. c) The graph in image provides the average particle distances within the shown assemblies in (a) for each sample. The scale bar in image (b) is 5 nm^−1^.

The presented results clearly demonstrate how the conductivity of the PtNC mesocrystals can be tuned solely by the choice of the capping agent, as there is a direct correlation between mesocrystal conductivity and stabilizer. A comparison of the molecular structures of the three used fatty acids which typically cap on the surface of the nanocubes shows their chemical similarity and only difference within the number of carbon–carbon double bonds (**Figure** **2**a). This minor structural variation however appears to have a significant impact on the conductivity within a highly ordered superstructure obtained from these particles. A physical interconnection between the individual nanoparticles within the assembly can be ruled out at this point due to their ability to be re‐dispersed in organic solvents. We, therefore, assume that the spatial arrangement of the PtNCs primarily dictates the ability to conduct electrons through the mesocrystals. In fact, the structural variation of the capping molecules on the PtNC surfaces causes an increase in the particle gaps, which we were able to observe through high‐resolution transmission electron microscopy (HR‐TEM) as the images in Figure 2a illustrate. To correctly measure the particle gap, we arranged the PtNCs of all three capping agents into 2D self‐assembled monolayers through solvent evaporation and determined the average facet‐to‐facet distance of several parallel oriented particle pairs as seen in Figure 2b. The particle arrangement within the monolayers follows a primitive cubic packing structure with a slight tendency toward a hexagonal ordering (Figure 2a) in some cases, as we have discussed in more detail in previous work.^[14b]^ HR‐TEM imaging at 200kX was combined with selected area fast Fourier transformation (FFT) of the particle arrays to further ensure correct particle orientation in regards to their facet‐to‐facet arrangement as shown in Figure 2b when measuring the size of the gap. In addition, the observable long‐range ordering of the atomic lattices throughout the particle arrangement further demonstrates the mesocrystallinity and therefore comparability of the obtained particle arrays with the bulk mesocrystals. The chart in Figure 2c shows how the average facet‐to‐facet particle distance increases alongside the number of double bonds of the fatty acid. When it comes to electron conductivity properties of 2D and 3D superlattices, the spatial distance between the nanoparticles is already known to be a key factor concerning the transfer of electrons.^[^ ^18^ ^]^ It is further evident that the lattice structure of the particle assembly can also have an influence on the particle distance or the cross section of the facet‐to‐facet interaction and therefore might affect electron propagation along a specific crystallographic orientation. In previous research, we showed that the lattice structure of the PtNC‐based mesocrystals from hexane show a high tendency toward a hexagonal packing in one plane and a rhombohedral crystal habitus.^[14a]^ Hence, we measured the resistance perpendicular and diagonal to the outer faces of the mesocrystals in an effort to rule out any secondary effects caused by the internal structure of the mesocrystal. As demonstrated in Supporting Information 3, we were not able to observe any clear difference in conductivity in relation to the orientation of the crystal.

Furthermore, a variation in the crystallographic arrangement of the PtNCs in mesocrystals from OLA, LOA, or LLA is unlikely given the results of preceding work where it is shown that the crystal structure of a gas‐phase diffusion self‐assembled mesocrystal is dictated by the solvent and stabilizer concentration rather than its number of C═C double bonds.^14^, ^19^ These findings conclude that the increasing facet‐to‐facet particle distance, which we examined in the 2D self‐assemblies, must be taking effect within the 3D mesocrystals as well and are the primary contributor to the decrease in conductivity. SAXS as the analytical tool of choice when it comes to determining particle distances within the bulk mesocrystal however cannot be used due to the varying particle sizes of the individual building units. Although the PtNC dispersions display a quite narrow size distribution, the PtNC sizes still vary within a couple of nanometers even after recrystallization, which is an order of magnitude difference in relation to the minor change in particle distance between different stabilizers. Therefore, it is impractical to compare the different mesocrystal batches solely based on SAXS. For this reason, the comparison of PtNC distances in a 2D assembly is a more accurate reference. In any case, these findings are concurrent with our prior conclusion that the electrical conductivity is primarily determined by the facet‐to‐facet particle gaps. Especially in the case of metal nanoparticle networks surrounded by insulating organic molecules, a thermally activated electron tunneling mechanism should dominate which causes an exponential dependence of the electrical conductivity to the interparticle distance.^[^ ^20^ ^]^ In addition, the property of the chosen PtNCs to preferably self‐orient into a facet‐to‐facet mesocrystalline lattice, as indicated in Figure 2b, has also been demonstrated in preceding research.^[14a]^ This concludes that the proposed tunneling mechanism is favored due to the increased surface area at which electron tunneling effects can occur.^[^ ^21^ ^]^

**Figure 3:**
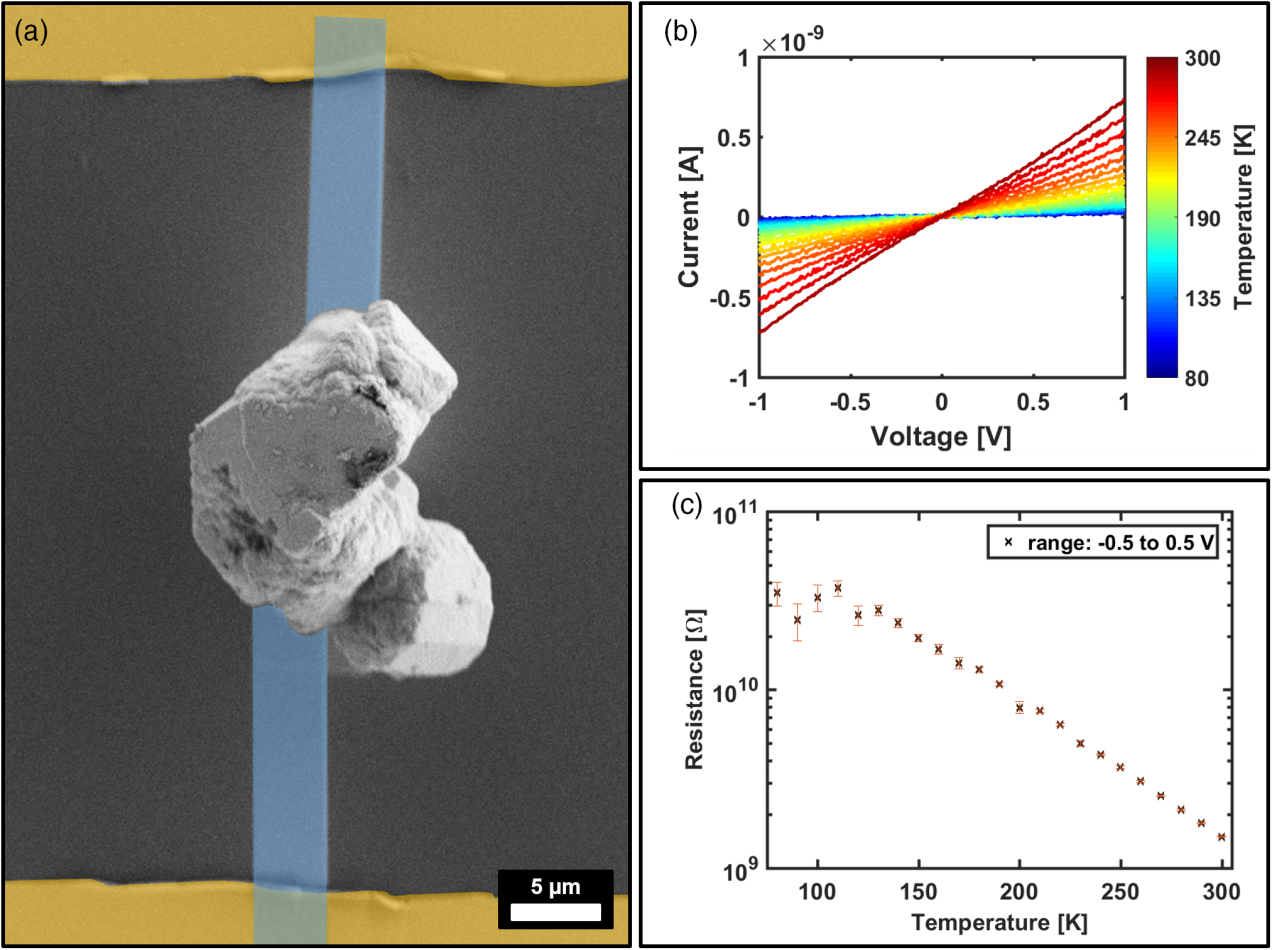
Temperature‐dependent *I–V* measurement of a single‐OLA‐stabilized PtNC mesocrystal. a) The FE‐SEM image shows the mesocrystal connected to prefabricated Au electrodes (shaded yellow) through deposited Pt lines (blue shaded). b) The graph illustrates how the measured current is increasing for elevated temperatures, which implies a thermally activated conduction mechanism. Therefore, the extracted low‐voltage resistances show c) an exponential dependence with the sample temperature.

To further verify a thermally activated electron tunneling model as the predominant conduction mechanism, the OLA‐stabilized PtNC mesocrystals were subjected to a temperature‐dependent conductivity measurement. Respective mesocrystals were transferred to a nonconductive substrate with prefabricated Au finger electrodes and contacted via focused ion beam (FIB)‐assisted Pt‐deposition (**Figure** **3**a). In the case of thermally activated electron tunneling, the conductivity of the mesocrystals should increase with elevated temperatures in contrast to a metallic behavior where an increased electron scattering will result in a reduction in conductivity.^[14e]^ The OLA specimen was cooled to a temperature of 80 K and then stepwise heated by 10 K to a temperature of 300 K with conductivity measurements in between every heating step. The graph in Figure 3b demonstrates how the *I–V* curves evolve during the experiment. For low temperatures, the mesocrystal is almost insulating with resistances near the resolution limit which is indicated by the increased error bar of extracted zero‐bias resistances (used range: −0.5 to +0.5 V) in Figure 3c. At a temperature of 130 K and upward, an exponential decrease in resistance with rising temperatures can be observed. These findings are in good agreement with the thermally activated electron tunneling model as the main contributor to the conduction mechanism in the as‐prepared mesocrystals. This likewise rules out a metallic behavior that might arise from a physical contact between the individual PtNCs inside the mesocrystal superlattice, that is, the PtNCs are separated by the organic ligand which dominates its electrical behavior.^[^ ^22^ ^]^

In contrast to this temperature‐dependent behavior, we also observed an irreversible increase in conductivity throughout for extended measurement cycles on a single crystal when the temperature is fixed at RT (Supporting Information 2, Figure S4b, Supporting Information). We assume that it is a result of a permanent altering of the mesocrystalline structure due to either induced Joule heating or interactions with the SEM electron beam as it only occurs after several measurements on the same mesocrystal. The soft and fragile texture of the mesocrystals outlines one major downside of these new types of materials. Recent research however has demonstrated a method on how OLA‐stabilized superstructures can be heat‐treated to organically interconnect the carbon–platinum framework resulting in enhanced structural properties.^[15a,15b]^ It is therefore worthwhile to investigate how this treatment will affect the electrical properties of the PtNC‐based mesocrystals in respect to the conduction mechanism discussed in this work. Hence, we heat‐treated all three mesocrystal types to induce a permanent altering of the entire samples through potential linkage of the fatty acids and subsequently investigated their electrical conductivity again.^15^, ^23^

**Figure 4:**
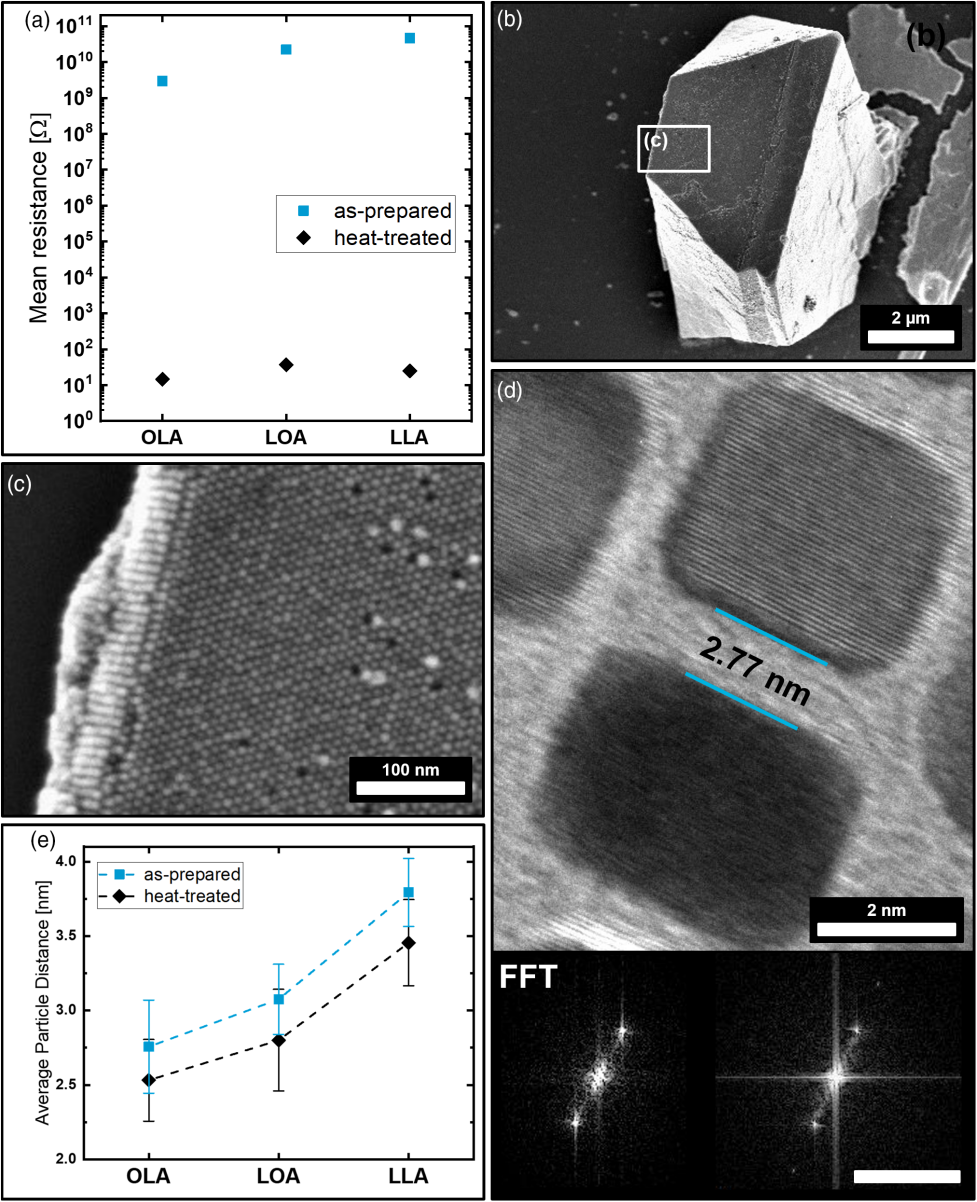
a) Graph compares the mesocrystal conductivities as determined from several specimens for each, heat‐treated and as‐prepared (untreated) sample. b, c) FE‐SEM images show a mesocrystal and its surface after heat treatment, indicating no significant altering of the mesocrystalline structure has occurred. d) The HR‐TEM image shows a heat‐treated LOA‐stabilized PtNC 2D self‐assembly with an exemplary determined particle distance of 2.77 nm as well as the corresponding FFT analysis to determine the particle orientation. e) Graph illustrates the measured average particle distances of heat‐treated and as‐prepared samples. Scale bar in (d, inset) is 10 nm^−1^.

The previously analyzed 3D mesocrystals were subjected to a heating ramp to 325 °C over the course of 3 h under a nitrogen atmosphere to slowly alter the fatty acids, which interconnect the individual PtNCs. Infrared spectroscopy of the heat‐treated mesocrystals in comparison to the untreated specimen shows an absence of the sharp C═C stretching modes as well as two emerging broad signals at 1050 and 3300 cm^−1^ (Supporting Information 4).^[^ ^24^ ^]^ This strongly suggests a significant decomposition of the OLA into a carbon framework most likely induced by the catalytic properties of platinum. Nonetheless, the mesocrystal rigidity for all three samples increased significantly as can be observed when applying force with the nanoprobing tips. The harder mesocrystal surface allowed for a high contact pressure, again ensuring a reliable nanoprobe contact to the measured samples. With this, we were able to first demonstrate how PtNC‐based mesocrystals stabilized by OLA, LOA, or LLA can be thermally hardened similar to iron oxide nanoparticle supercrystals.^[15a,15b]^ Surprisingly, a drastic increase in electrical conductivity by at least seven orders of magnitude after heat treatment can be recorded for all three types of mesocrystals (**Figure** **4**a). With a mean resistance of only 15, 37, and 25 Ω for five measured OLA, LOA, and LLA crystals, respectively, the mesocrystals show a metallic conductivity as the measured resistance is within the range of the inherent resistance of the used nanoprobing system. The drastic increase in conductivity can no longer be explained by a thermally activated tunneling of electrons and rather suggests a direct contact between the nanoparticles that emerged after heat treatment. FE‐SEM imaging of the heat‐treated mesocrystal samples, however, does not indicate a significant alteration in their structure as Figure 4b,c reveals. Corresponding to the reference experiments on the 2D PtNC arrays for the untreated samples, further insight on how the heat treatment will affect the nanoparticles and its stabilizer on the nanometer scale can be obtained by the means of HR‐TEM. Therefore, all three particle types were assembled through solvent evaporation on a 15 nm silicon nitride membrane and heat‐treated at 325 °C analogous to the mesocrystal specimen. The organically linked 2D assemblies were analyzed by HR‐TEM and FFT to determine their facet‐to‐facet particle distance and orientation as illustrated in Figure 4d. In addition, no sign of PtNC altering, deformation, or particle fusion could be observed for the 2D assemblies. While the trend of the particle spacing from OLA to LLA remains identical to the as‐prepared PtNCs, it is evident how the particle distance before and after heat treatment notably reduced by over 8% from 2.8 ± 0.3 to 2.5 ± 0.3 nm for OLA, 3.1 ± 0.2 to 2.8 ± 0.3 nm for LOA and 3.8 ± 0. 2 to 3.5 ± 0.3 nm for LLA in all three cases as illustrated in Figure 4e. This change in particle distance suggests an increase in conductivity due to a smaller tunneling barrier at shorter particle distances for the mesocrystals but does not solely explain an increase of seven orders of magnitude.

**Figure 5:**
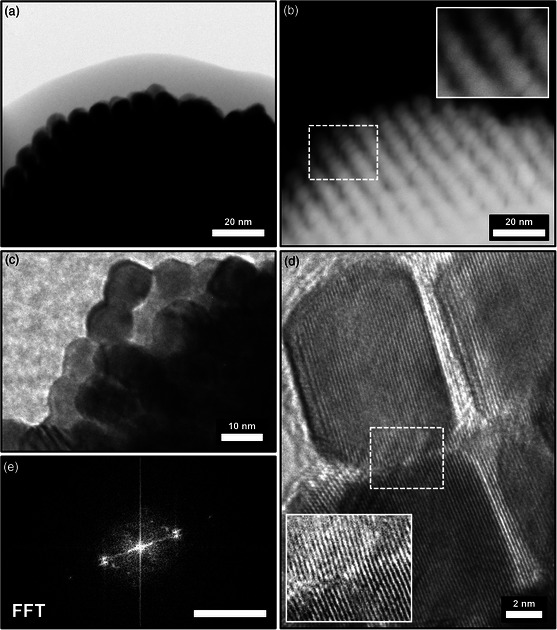
Scanning transmission electron microscopy (STEM) images in a) transmission bright field and b) high‐angle annular dark field (HAADF) of a heat‐treated OLA‐stabilized PtNC mesocrystal fragment. HR‐TEM images at 300 kX show c) the partially fused and d) mineral‐bridged PtNCs forming a highly crystalline structure as e) the FFT of image (d) reveals. Scale bar in (e) is 5 nm^−1^.

Hence, HR‐TEM in combination with high‐angle annular dark‐field scanning transmission electron microscopy (HAADF‐STEM) imaging on the heat‐treated mesocrystals was performed to fully explain this behavior. Due to the strong electron absorption of platinum, a thin piece of a fragmented mesocrystal sample was investigated which allows transmission of electrons and the observation of the inner mesocrystal rather than its exterior surface (**Figure** **5**a,b). Several rows of ordered PtNCs with small but visible gaps in between can be located. The nanocubes appear to have retained their cubic shape and order, although their gaps are much narrower often contacting neighboring particles in at least one direction of ordering as illustrated in Figure 5b inset. HR‐TEM images at 300kX shown in Figure 5c,d reveal how mineral bridges and particle fusion of the individual PtNCs occurred in parts of the mesocrystal. Figure 5d and the corresponding FFT analysis (Figure 5e) demonstrate how the initial mesocrystalline particle orientation shown earlier facilitates particle fusion of the PtNCs into a single crystalline superstructure. The formation of mineral linkage and particle fusion upon heating in semiconductor nanoparticle‐based mesocrystalline films on the example of PbS nanoparticles has been reported before^[^ ^25^ ^]^ supporting our observations. Furthermore, the thermal altering of an OLA into a carbon framework as we observed it (Supporting Information 4) is already known to promote electron transport, which was shown on the example of self‐assembled Fe_3_O_4_ nanocube superlattices.^[15b]^ Therefore, we conclude that the formation of mineral bridges between the particles is the primary reason for the high conductivity of the heat‐treated mesocrystals, supported by a contributing effect of the altered carbon framework enhancing its conductivity even further. Although the formation of mineral bridges most likely dominates the electrical conductivity throughout the mesocrystal it remains unclear how high the contribution of electron propagation through the carbon framework or tunneling effects is. In an effort to reliably quantify the individual effects on overall conductivity in future work, it will be necessary to separate the carbon framework from the platinum structure through oxidation/dissolving. In addition, sophisticated high‐resolution 3D mapping of the mesocrystal could be conducted, to quantify the degree of mineral bridge formation within the bulk of the crystal.

In an effort to expand and compare our findings to other mesocrystalline superstructures, we conducted measurements on mesocrystals fabricated from iron oxide nanocubes (IONCs) of a similar size (12.0 ∓ 1.5 nm) and likewise stabilized by OLA in hexane (Supporting Information 5). It is important to notice here that the IONCs consist of a mixture of predominantly Fe_3_O_4_ (magnetite) with minor amounts of Fe_2_O_3_ (maghemite).^[9b]^ As expected, we found that the resistance of these mesocrystals reached the limits of the nanoprobe measuring system as a result of the low inherent conductivity of magnetite/maghemite in combination with the high barrier of electron tunneling due to OLA (Figure S8b, Supporting Information). With a resistance of >100 GΩ, it can be stated that the IONC‐based mesocrystals are highly insulating. Analogous to the PtNC‐based mesocrystals, the IONC‐based mesocrystals were heat‐treated and subsequently measured to exhibit a significantly lower resistance of 32 ± 3 MΩ (Figure S8c, Supporting Information). This value is in good agreement with the reported conductivity for bulk of a magnetite/maghemite mixture and when additional discontinuities within the material are considered.^[^ ^26^ ^]^ This further consolidates our previous findings that demonstrate the formation of mineral bridges allowing electron propagation comparable to the bulk material.

**Figure 6:**
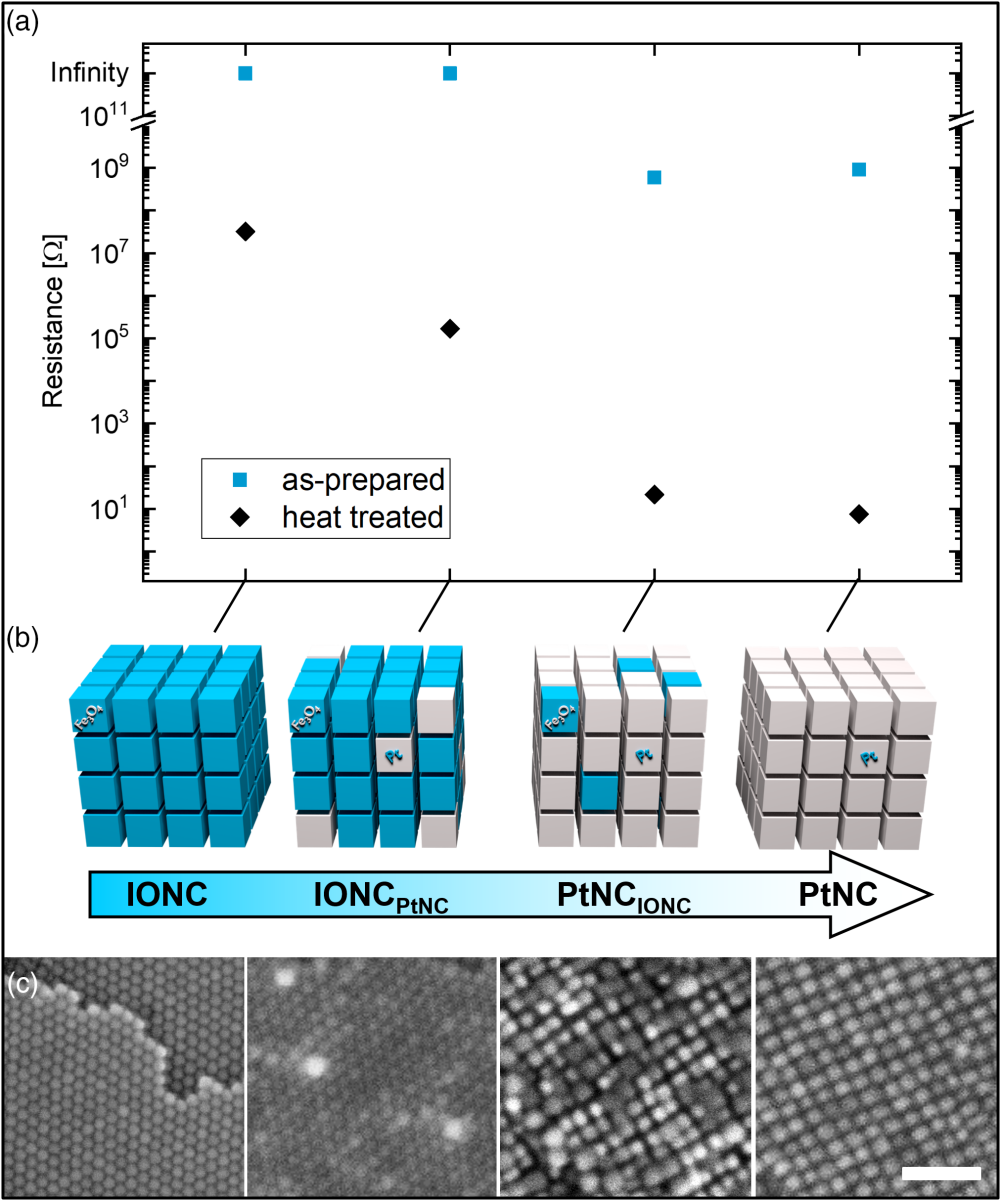
a) The resistances of monophase PtNC‐ and IONC‐based mesocrystals are compared as well as binary PtNC/IONC mesocrystals (blue) with their heat‐treated analogues (black). Resistances have been obtained through the previously described *I–V* measurements on a sample size of five mesocrystals per type. b) All four different types of mesocrystals are illustrated indicating their IONC/PtNC content from left to right. c) FE‐SEM images of the mesocrystal surfaces allow for a differentiation of high‐contrast PtNC from low‐contrast IONC due to an increased secondary electron emission for high atomic number materials. Scale bar in (c) is 50 nm.

While platinum nanoparticles are already well known for their catalytic and electrochemical properties, iron oxide nanoparticles also hold astounding new properties such as superparamagnetism.^[9a]^ To expand the observations made in this work onto multicomponent materials, we utilized both materials to create micrometer sized 3D binary mesocrystals that contain PtNCs as well as IONCs according to our recent work.^[14b]^ The obtained structures are illustrated in **Figure** **6**b and have been analyzed in Supporting Information 6. Upon determining their conductivity, we found that the incorporation of foreign particles into a mesocrystalline host lattice (Figure 6c) does not result in a measurable impact on the conductivity of the untreated binary mesocrystals. Binary mesocrystals where PtNCs form the host lattice and IONCs are incorporated into the structure (PtNC_IONC_) exhibit a similar conductivity (595 ± 61 MΩ) as pure PtNC‐based mesocrystals with a resistance of 915 ± 92 MΩ (Figure 6a). Equal observations can be made for the inverse counterpart where PtNCs are incorporated into an IONC mesocrystal host lattice (IONC_PtNC_). Again, embedding of PtNCs does not result in a measurable increase in conductivity of the mesocrystals as they still show the insulating behavior of pure IONC based mesocrystals (Figure 6a). In reference to our preceding work, where we analyzed the portrait binary mesocrystal samples, we demonstrated that a significant amount of “foreign” particles are embedded into the host mesocrystals by the means of FE‐SEM, energy dispersive X‐ray (EDX), and HR‐TEM analysis (Figure S9, Supporting Information) as well as software‐assisted particle detection methods.^[14b]^

When it comes to the conductivity of the heat‐treated binary specimen, a slightly different picture is painted. Our findings so far demonstrated that the heat treatment resulted in a formation of mineral bridges between the individual building blocks. Thus, a physical connection between the different materials can be expected, which should result in a contribution of the introduced particles to the electrical properties of the host material. In fact, we found that the resistance of the mesocrystals actually behaves as one would expect when mixing a highly conductive material with an insulating one. The conductivity of IONC_PtNC_ binary mesocrystals increases significantly by two orders of magnitude with a measured resistance of 168 ± 17 kΩ. In accordance, an inverse effect is measurable for the PtNC_IONC_ binary mesocrystals. There, an increase in resistance from 7.5 ± 0.8 to 22 ± 2 Ω is measurable, yet less pronounced in comparison to its inverse counterpart. Based on these observations, the amount of incorporated particles, which had been determined to be only 0.68% for IONC_PtNC_ but significantly higher for PtNC_IONC_ binary mesocrystals, appears to directly correlate to the conductivity of the material.^[14b]^ Although the remaining obstacle of precisely controlling the amount of incorporated particles within the binary mesocrystals needs to be overcome first, this method already allows for a continuous tuning of the conductivity as a function of amount of incorporated particles.

Nevertheless, we were able to provide analytical evidence that this method is a cost‐effective way to increase the platinum mesocrystal volume without diminishing its electrical conductivity for the untreated samples or even tune it if subsequently heat‐treated. This enables the introduction of potential catalytic, magnetic, or even superparamagnetic properties into the otherwise monophasic mesocrystal by the combination of two materials. The exact physical and electrochemical effects however have to be investigated in more detail to clarify any dependencies. Ultimately, a much more sophisticated analytical process including synchrotron‐based SAXS and WAXS measurements, SQUID measurements, as well as nanometer resolution element mapping is needed to conclude any well‐grounded explanations. The latter section of this research article therefore focused on presenting a brief outlook into the vast possibilities, which come alongside such multicomponent superstructures that can be explored in further work.

## 3 Conclusion

Within the scope of this work, we presented a method to determine and investigate the electrical properties of colloidal metal/metal oxide superstructures in form of large, micrometer‐sized PtNC‐ and/or IONC‐based mesocrystals. Conductivity measurements were established through nanoprobing individual specimen and recording multiple successive *I–V* sweeps. Through variation of chemically similar but structurally different PtNC capping agents in form of OLA, LOA, and LLA, we can reproducibly tune the conductivity of our mesocrystals. In an effort to study the underlying electron conduction mechanism, we observed that the inherent mesocrystal resistance increases alongside the facet‐to‐facet particle gaps within the assemblies. This change in particle distance is caused by the varying amount of C═C double bonds within the capping agent as we report. In addition, temperature‐dependent *I–V* measurements rule out a metallic conduction mechanism as the mesocrystal resistance reduces exponentially with increasing temperatures. As a result, we conclude that a thermally activated tunneling mechanism is responsible for the electron propagation throughout the mesocrystal. This mechanism is furthermore favored by the mesocrystalline ordering that facilitates an oriented facet‐to‐facet particle arrangement and suggests that the observed conduction mechanism should therefore be transferable onto many other metal nanoparticle‐based mesocrystals. The deliberate selection of unsaturated fatty acids as the intermediate surfactant further enables a hardening of the soft and fragile mesocrystal structure through organically linking while simultaneously increasing the conductivity by seven orders of magnitude. Furthermore, we were able to show that our findings can also be expanded onto multicomponent systems such as binary mesocrystals, where the interplay of platinum and iron oxide nanoparticles can lead to multifunctional materials. It was clearly demonstrated that the electron‐conducting behavior of such materials can either be retained or be tuned by subsequent heat treatment while adding the magnetic or potentially superparamagnetic properties of iron oxide nanoparticles.

The broad range of properties from soft to hard, dissolvable to permanent, and insulating to conducting that can be achieved with this novel class of metamaterial is highly promising. This demonstrates how nanocrystals can act as a versatile building block for a potential fabrication of functional mesocrystals with controllable electronic features.

## 4 Experimental Section

4.1

4.1.1

##### Chemicals

Tungsten hexacarbonyl (99%) and platinum (II) acetylacetonate (98%) were provided by abcr (Karlsruhe, Germany). LOA (99%), toluene (99.8 + %), and oleylamine with a C‐18 content of 80–90% were purchased from Acros Organics (Geel, Belgium). LLA (70%) was supplied by TCI (Tokyo, Japan) and OLA (99%) was received from Alpha Aesar (Kandel, Germany). Tetrahydrofuran (100%), hexane (98%), and ethanol (99,8 + %) were purchased from VWR (Darmstadt, Germany) as well as Roth (Fontenay‐sous‐Bois, France). All chemicals were used without further purification.

##### Platinum Nanocube (PtNC) Synthesis

The synthesis of platinum nanocubes were carried out according to a slightly modified procedure, which has been reported by Zhang et al.^[14c]^ A mixture of 3.56 g of OLA in 16 mL of oleylamine and 40 mg platinum(II) acetylacetonate was added and placed in a two‐neck Schlenk flask equipped with a reflux condenser, attached to a N_2_‐Schlenk line. The mixture was heated, subjected to vacuum for 10 min and subsequently heated to 120 °C under a slow nitrogen flow while vigorous stirring. Tungsten hexacarbonyl of 100 mg were added to the solution and the temperature was raised to 245 °C. This temperature was held for further 60 min under continuous agitation before cooling back to room temperature. The crude black product was separated by centrifugation with a relative centrifugal force (RCF) of 7960 G for 25 min and treated with anhydrous hexane in three separation cycles. Platinum nanocubes were obtained as a black and oily solid and redispersed in either hexane, toluene, or tetrahydrofuran for further use. The particle dispersion was stored under light exclusion to prevent decomposition.

##### IONC Synthesis

A heating‐up method was used to produce IONC from an Iron (III) Oleate Precursor in a two‐step synthesis.

To obtain the Iron (III) Oleate Precursor, a 100 mL two‐neck Schlenk flask was filled with Iron (III) chloride hexahydrate (1.35 g, 5 mmol) and sodium oleate (4.58 g, 15 mmol) under nitrogen atmosphere, degassed, and subsequently stirred in a mixture of ethanol (10 mL), hexane (18 mL), and MilliQ water (8 mL) until everything dissolved. This solution was freeze–pump–thawed in a liquid nitrogen bath for one cycle to remove any solved oxygen. While stirring, the mixture was heated to a reflux temperature of 80 °C for 4 h. The resulting two phase product was washed with milliQ water (3 × 50 mL) and the organic phase separated before drying over Mg(SO_4_)_2_ to remove any water residues. Rotary evaporation at 50 °C/330 mbar was performed to remove the organic solvents, until the crude product in form of a dark brown oil was obtained, which is stored at 4 °C until further use.

The previously prepared iron (III) oleate (4.54 g, 5 mmol), sodium oleate (218 mg, 0.72 mmol) and highly purified fatty acid (e.g., 99% pure OLA) (227 μL, 0.72 mmol) were placed within a three‐neck flask and dissolved in 1‐octadecene (25 mL). The mixture was then dried under vacuum at 60 °C for 30 min while stirring to remove remaining water and dissolved oxygen. The so‐prepared reaction mixture was subjected to a reflux temperature of 320 °C under nitrogen atmosphere using a heating ramp of 3.3 °C min^−1^. At this temperature, the reaction was stirred for further 30 min before removing the heating source and cooling back to room temperature while stirring. The now black liquid was transferred into several falcon tubes and purified through centrifugation for multiple times. At first, double the amount ethanol was used to dilute the crude product and separated with an RCF of 210 G (15 min, two times) and then with a mixture of ethanol to toluene decreasing each washing cycle (4:1, 3:1, 2:1) for three times at an RCF of 9000 rpm (15 min). Larger agglomerates could be separated by a syringe filter after dispersing the purified product in the desired solvent such as tetrahydrofuran (THF), toluene or hexane.

##### Mesocrystal Formation via Gas‐Phase Diffusion

A cleaned 5 × 7 mm double‐side polished silicon wafer snippet and 300 μL of a prepared particle dispersion containing 3 μL mL^−1^ of highly purified fatty acid (e.g., OLA [99%]) were placed within a 1 mL flat bottom glass vial. The silicon snippet was cleaned by 10–15 min gradual ultrasonification in first ethanol then isopropanol, acetone, ethyl acetate, toluene, and toluene. The so‐prepared 1 mL flat bottom glass vial was placed inside a 5 mL screw cap vial, which was prepared to contain 1.5 mL of an ethanol/dispersion solvent (50:50) mixture. The crystallization setup was then stored within a desiccator containing an ethanol‐rich atmosphere for multiple days, depending on the dispersion solvent (THF 1–2 days, toluene 7–14 days, hexane 14+ days). When the crystallization was finished, the mesocrystals holding silicon snippet was carefully removed and immediately immersed into a pure ethanol solution for 30 s before drying in air.

##### Binary Mesocrystal Formation via Gas‐Phase Diffusion

Binary mesocrystals were prepared analogous to the formation of monophase mesocrystals as described earlier. The prepared particle dispersions however consisted of mixtures of equally sized particle batches from two different materials (e.g., IONCs and PtNCs) as determined via TEM analysis (Supporting Information 1 and 5). The mixing ratios can vary depending on other factors such as particle concentration as well as particle size distribution and need to be determined in various control experiments. The resulting particle dispersion mixture was prepared to contain 3 μL mL^−1^ of highly purified fatty acid (e.g., OLA [99%]) before continuing with the mesocrystal formation.

##### Heat Treatment of Fatty Acid–Stabilized Mesocrystals

The prepared mesocrystal samples were heat‐treated in a nitrogen atmosphere using a Unitemp rapid thermal process (RTP) oven. Prior to heating, the oven was evacuated and flushed with nitrogen gas three times, before slowly raising the temperature to 320 °C over the course of 3 h. The temperature was then held for further 20 min before slowly cooling to room temperature within 1 h. Heat‐treated mesocrystals show a significant change in structural rigidity as already reported in literature.^[15a]^

##### Sample Fabrication

*p*‐doped silicon (Si) substrates with a 1000 nm (±5%) thick thermally grown silicon dioxide (SiO_2_) layer from Active Business Company GmbH were cut into 5 × 7 mm pieces and cleaned in deionized water, followed by acetone and isopropanol in an ultrasonic bath for 10 min each. An optical lithography process in combination with reactive ion etching (RIE) was applied to generate a marked grid with 300 × 300 μm fields, which enabled to locate a specific mesocrystal. For mesocrystal formation, the samples were placed in the 1 mL glass vial mentioned beforehand.

##### In Situ Nanoprobing

For in situ *I–V* measurements, a nanoprobing system from Imina Technologies was transferred to the SEM chamber, which enabled the usage of up to four individual miBot nanoprobers. Tungsten tips with a radius of 100 or 1000 nm were used for measurements. A well‐grown mesocrystal per marker field was selected and contacted by two of the tips. The nanoprobes were connected to a Keithley 2401, which could be controlled remotely by a MATLAB program to perform in situ *I–V* measurements (voltage range: from 1 μV to 20 V, current range: from 10 pA to 1 A). For every sample, 5 to 10 different as‐prepared and heat‐treated mesocrystals were measured and the distance between the tips was kept between 4 and 10 μm dependent on mesocrystal size. The electron beam was blocked during electrical measurements to prevent influences of incident electrons.

##### Temperature‐Dependent I–V Measurements

Grown mesocrystals were transferred to a Si wafer with a 1 μm thick SiO_2_ layer on top for temperature‐dependent *I–V* measurements. The sample exhibits prefabricated finger electrodes with distances of 20 and 40 μm. Afterward, a nanoprobe manipulation is used to deposit a single mesocrystal between the finger electrodes. For fixation and contacting of the crystal, an FIB in combination with a gas injection system (GIS) was applied to deposit Pt connections. The sample was placed in a cryostat to perform *I–V* measurements with a Keithley 2401 from 80 to 300 K in vacuum (pressure <10^−5^ bar). To ensure equilibrium, the sample was kept at each temperature for 15 min before performing an *I–V* sweep.

## Conflict of Interest

The authors declare no conflict of interest.

## Supporting information

Supplementary Material

## Data Availability

The data that support the findings of this study are available from the corresponding author upon reasonable request.
