# Supplementary material for: Tuning the Electronic Properties of Mesocrystals
Source: Small Sci. 2022 Jun 28;2(8):2200014. doi: 10.1002/smsc.202200014 (PMC11935787; doi:10.1002/smsc.202200014)
Supplement: Supplementary file 1 — Supplementary Material [file SMSC-2-2200014-s001.pdf]

Supporting Information

Supporting Information: Tuning the Electronic Properties of Mesocrystals

Christian Jenewein[†], Stefan M. Schupp[†], Bing Ni, Lukas Schmidt-Mende, Helmut Cölfen**

Supporting Information 1: Platinum Nanocubes (PtNCs)

We used TEM imaging in combination with a software (Olympus iTem) assisted particle detection method to determine the number weighted average particle size of our platinum nanocube dispersions as well as their size distribution and aspect ratios as shown in **Table S1** and **Figure S1**. The center (x_c) of the Gaussian fit gives the average particle while we chose the Full Width at Half Maximum (FWHM) to represent the width of the size distribution, which we indicated by its value placed behind the ∓ symbol. The aspect ratio was determined for each particle individually and the arithmetic mean for the examined sample calculated.

Table S1. Particle analysis of three different stabilized PtNC dispersions. The center of the Gaussian fit (x_c) and its FWHM, as well as the arithmetic mean of the particle Aspect Ratio ($diameter_{max}/diameter_{min}$) of all three PtNC batches is shown in the Table below. The Total Count of particles detected for this analysis is shown in the last column.

PtNC Sample	x_c [nm]	FWHM [nm]	Aspect Ratio	Total Count
OLA	10.63 ± 0.01	2.25 ± 0.03	1.32 ± 0.11	1957
LOA	10.75 ± 0.09	2.48 ± 0.27	1.32 ± 0.19	1059
LLA	12.25 ± 0.10	2.96 ± 0.33	1.30 ± 0.20	2948

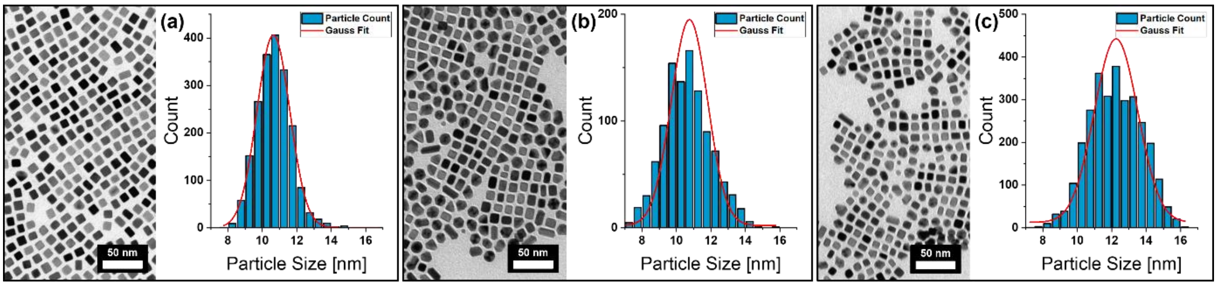

Figure S1. TEM images and the corresponding size distributions determined by software (Olympus iTem) assisted particle detection of OLA (a), LOA (b) and LLA (c) stabilized PtNCs.

Supporting Information 2: In-situ Nanoprobing of Individual PtNC Based Mesocrystals

The used Si/SiO_2 samples were structured before mesocrystal growth. Therefore, an optical lithography step in combination with an etching process was applied to generate a grid consisting of 15 (labeled with I-XV) 300 µm x 300 µm fields as depicted in **Figure S2a**. This enables the identification and several two-point I-V measurements of the same mesocrystal by the nanoprobing system, which is integrated in a FE-SEM, to investigate influences of different treatments and measurement conditions (**Figure S2b**).

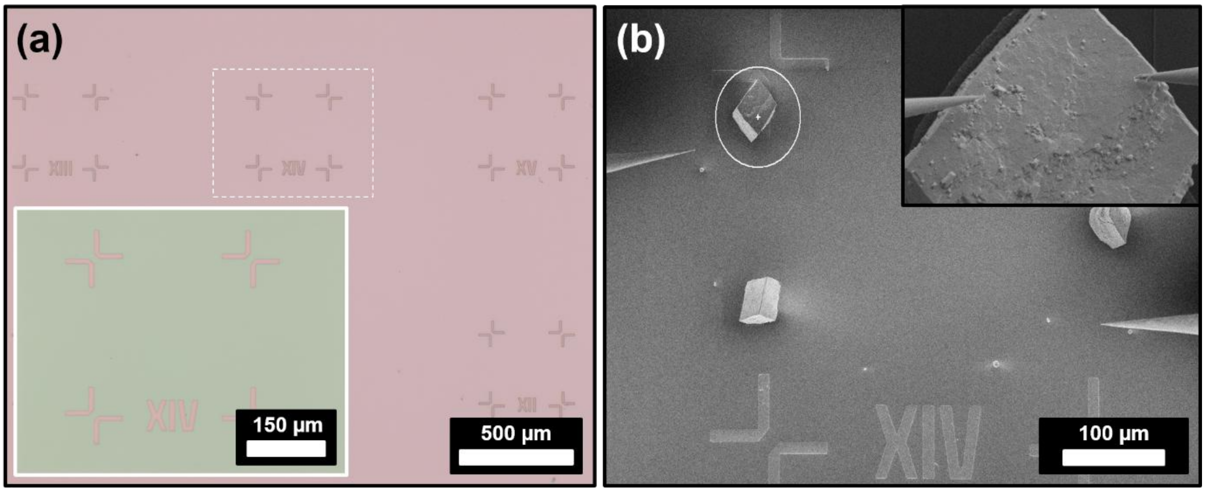

Figure S2. Mesocrystal selection for two-point measurements of single Pt mesocrystals. (a) An etched grid is used to identify coordinates of the selected mesocrystals. (b) Taken FE-SEM images allow the relocation of the same crystal to perform I-V measurements after different treatments.

However, I-V measurements on LOA (**Figure S3a and S3b**) and LLA (**Figure S3c and S3d**) stabilized mesocrystals with a tip distance of ~ 10 µm show an almost insulating behavior with resistances > 10^{10} Ω, which is in the range of the resolution limit of the used Keithley 2401. Therefore, the measured I-V curves are quite noisy resulting in an increased error of the calculated resistance values. Nevertheless, crystals with LLA are still less conductive than the LOA ones since the error is < 10 % of the determined resistances.

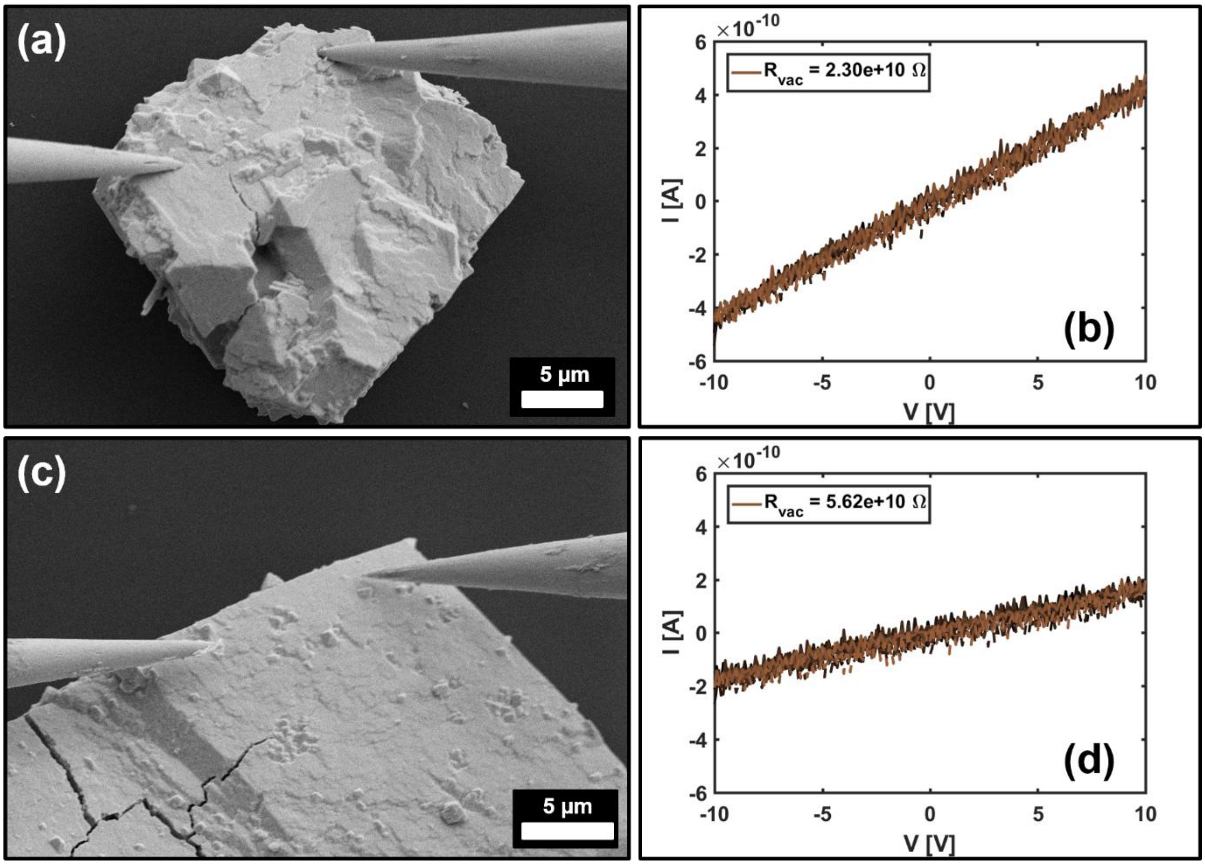

Figure S3. I-V measurements of LOA ((a) + (b)) and LLA ((c) + (d)) stabilized Pt mesocrystals. Both types of crystals show a very high resistance, which results in quite noisy I-V curves.

Regarding two-point measurements it is important to investigate the influence of contact resistance. To account for this, the same mesocrystals have been measured with different Tungsten tip diameters, namely 200 nm and 2000 nm. Consequently, the contact area should be roughly 100 times larger for the bigger tips and reduce the contact resistance drastically. Though, measured resistances with the 2000 nm tips (dark blue bars) are comparable to the 200 nm ones (light blue bars) as shown in **Figure S4a**. This result is further confirmed by test measurements on another mesocrystal per location with the bigger tips (green bars). Therefore, the contact resistance seems not to be a limiting factor and measured resistances reflect the electrical properties of Pt mesocrystals. However, it was observed that longtime measurements on the same mesocrystal can change its electrical properties quite drastically. Their conductivities increased irreversibly, which we attributed to a permanent altering of the

crystals itself resp. thermal crosslinking of the unsaturated fatty acids due to Joule Heating during I-V sweeps or electron bombardment of the electron beam. An example is shown in **Figure S4a** for the mesocrystal at position 14 which was measured with both tip diameters. The ratio R/L decreased more than two orders of magnitude after several longtime measurements. This increase in conductivity is also visible for the measured I-V curves at different measurement days in **Figure S4b**.

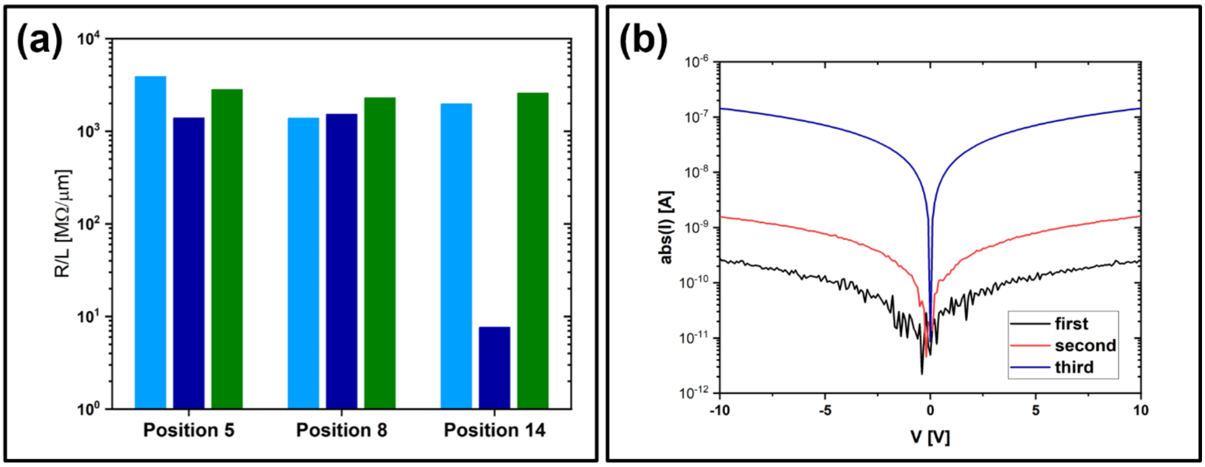

Figure S4. Influence of tip diameter and repeated measurements on the measured crystal resistance. (a) Measurements of the same mesocrystal by nanoprobe tips with a diameter of 200 nm (light blue) and 2000 nm (dark blue) show that the contact resistance is not a limiting factor. Another crystal measured with the 2000 nm tips (green) confirms this finding with a comparable resistance. However, if several extensive measurements are performed on a single crystal the resistance changes quite drastically (crystal at position 14), which is also observable in the I-V curves measured at different days as illustrated in (b).

Finally, the influence of tip placement is investigated. Usually, the nanoprobers are placed on the crystal surface to ensure a reproducible tip separation of ~10 µm and sufficient contact for different measurements. However, this could imply that the performed two-point measurements only reflect the conductivities of Pt mesocrystal surfaces instead of the bulk. To rule that out an OLA stabilized mesocrystal was transferred to a sample with pre-fabricated Au electrodes and the measurement tips were placed on the mesocrystal and electrode surface, respectively (**Figure S5a**). The resulting I-V curve and calculated resistance

in **Figure S5b** is comparable to previous measurements of this crystal type. Therefore, the performed two-point measurements on the crystal surface also reflect the intrinsic electrical properties of the 3D structure.

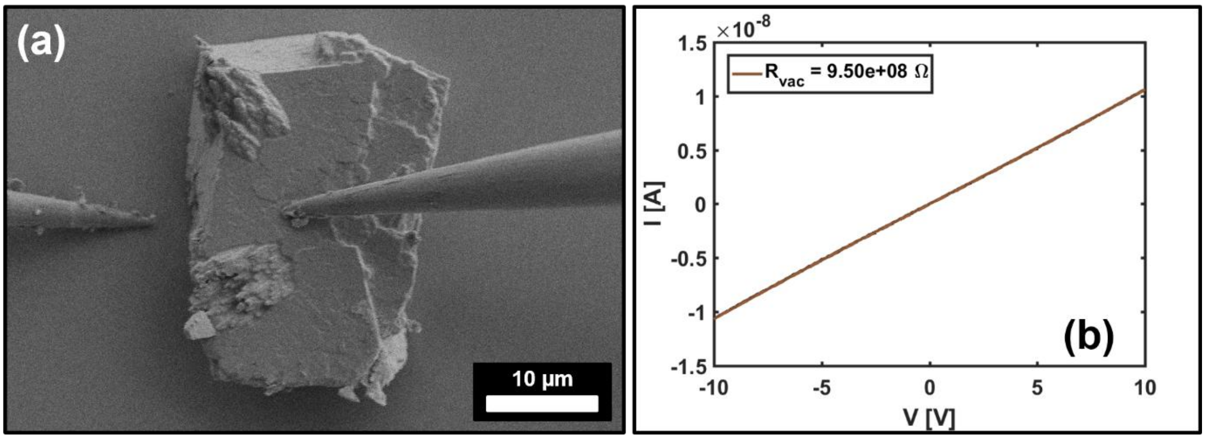

Figure S5. Influence of measurement direction. (a) A OLA stabilized crystal is placed on pre-fabricated Au electrodes to enable an I-V measurement through the crystal. (b) The I-V curve and calculated resistance is comparable to previous two-point measurements on the crystal surface.

Supporting Information 3: Conductivity Measurements along Mesocrystal Facets

Since the electrical conductivity of mesocrystals is dominated by the facet-to-facet particle distance the arrangement of nanoprobing tips in respect to nanoparticle orientation could influence measured resistances R. However, I-V measurements perpendicular and diagonal to the outer faces of OLA stabilized mesocrystals for a tip separation of L ~ 10 µm do not show a significant difference (**Figure S6**). Especially, the ratios R/L end up in similar values of 180 MΩ/µm and 181 MΩ/µm for measurements diagonal and perpendicular to the nanocube facets, respectively. Therefore, we could not observe any clear relation between the internal structure of mesocrystals and measured resistances.

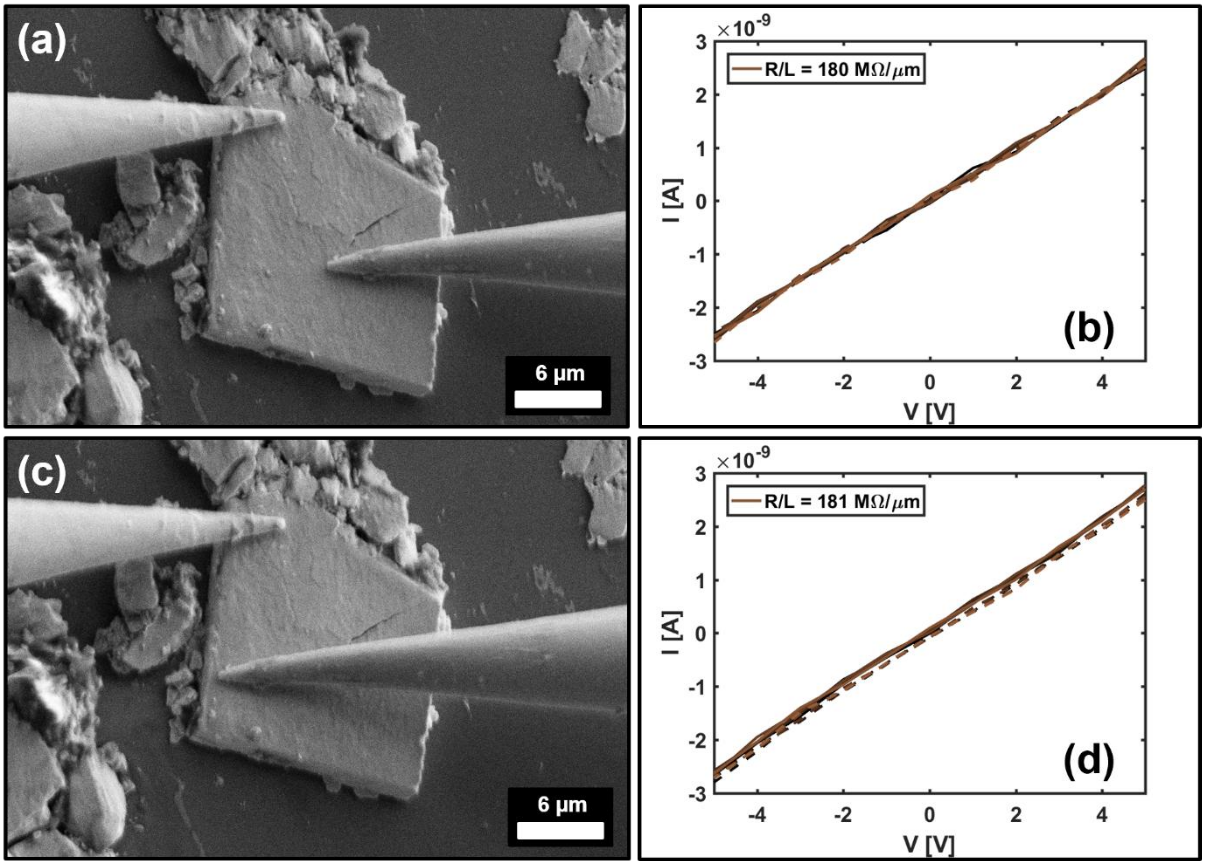

Figure S6. Influence of nanoparticle orientation. I-V measurements diagonal ((a) and (b)) and perpendicular ((c) and (d)) to the outer faces of the mesocrystal give similar ratios R/L, i.e. the influence of nanoparticle orientation on measured resistances seems to be negligible.

Supporting Information 4: ATR FT-IR Spectroscopy of PtNC based Mesocrystals

ATR FT-IR Spectroscopy of as-prepared OLA stabilized PtNC based mesocrystals was performed and compared with the spectra obtained from a heat-treated sample in order to investigate the altering of the oleic acid. In the as-prepared spectra of **Figure S7** two signals at 2920 cm^{-1} and 2852 cm^{-1} are visible which correspond to the CH_2 and CH_3 stretch modes of the OLA tail. The three sharp signals can be contributed to the C=C (1634 cm^{-1}) and C=O (1538 cm^{-1}) stretching as well as the C-H bending (1465 cm^{-1}) modes. The shift of the C=C and C=O stretching modes towards lower wavenumbers when compared to pure oleic acid is a result of the interaction of the COOH head group of the OLA with the PtNC surface.[24a] If the spectrum is compared to the heat-treated sample, it is evident how the CH stretching modes practically disappear and the C=C/C=O modes significantly decrease in strength

accompanied by a broadening of their signal. In addition, two broad signals at 3300 cm^{-1} and 1050 cm^{-1} emerge, indicating a strong decomposition of the OLA.[24b]

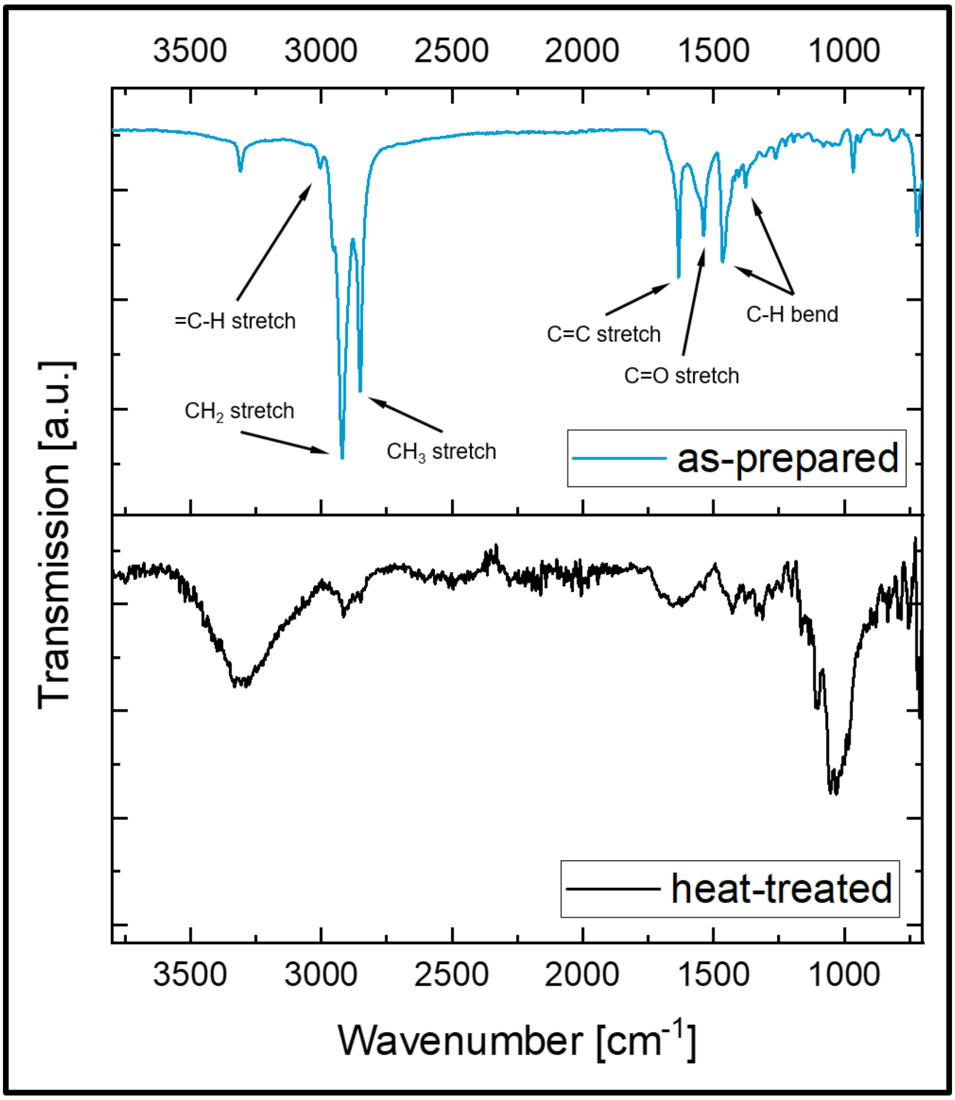

Figure S7. FT-IR spectra of an as-prepared OLA stabilized PtNC based mesocrystal in blue and the same specimen after heat-treatment at 325°C in black. The characteristic vibration modes have been labeled accordingly and their values are as follows: =C-H stretch (3007 cm^{-1}); CH_2 stretch (2920 cm^{-1}); CH_3 stretch (2852 cm^{-1}); C=C stretch (1634 cm^{-1}); C=O stretch (1538 cm^{-1}); C-H bend (1465 cm^{-1}).

Supporting Information 5: Iron Oxide Nanocube (IONC) Based Mesocrystals

Iron oxide nanocubes have been synthesized according a two-step heating method as it has been described in the experimental section and already reported in literature.[16a] The particles where characterized by the means of SAED and their average particle size was determined to be 12.0 ∓ 1.5 nm after re-crystallization through software assisted TEM analysis (**Figure**

S8a) analogous to the method described within Supporting Information 1. From these particles, mesocrystals were formed through the gas-phase diffusion technique to obtain superstructures of 50 to 100 µm in size. Conductivity measurements of the IONC based mesocrystals show a very high resistance of > 100 GΩ, which is above the detectable limit of the used nanoprobing system, indicating an insulating material as shown in **Figure S8b**. The crystals have been subjected to heat treatment equal to the PtNC based mesocrystals which resulted in an increase in conductivity and a resistance of 32 ± 3 MΩ (**Figure S8c**) which is close to the expected resistance of polycrystalline bulk iron oxide.[26]

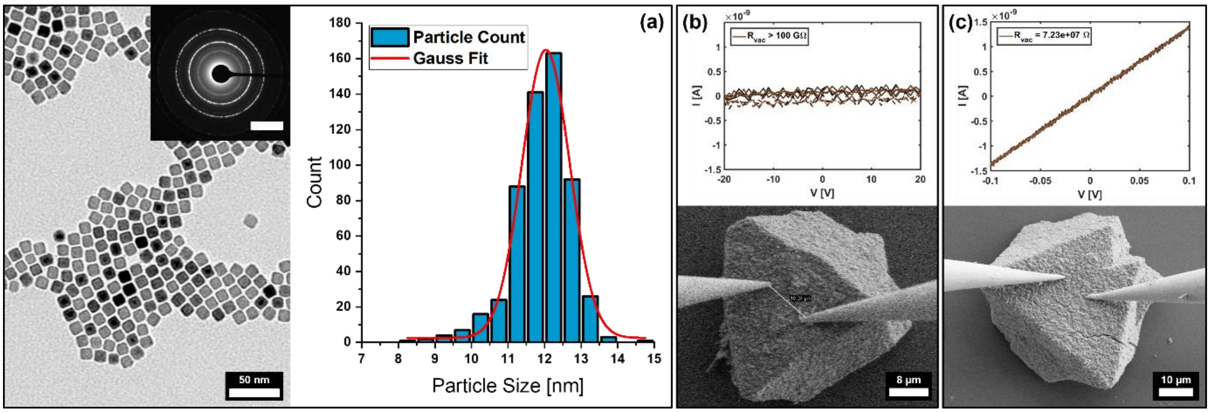

Figure S8. Software (Olympus iTem) assisted particle detection of TEM images of a recrystallized IONC batch gave an average particles size of 12.0 nm, which is indicated by the center x_c auf the Gaussian fit (red) in figure (a) and a particle size distribution of 1.5 nm given by its FWHM. FE-SEM images and the corresponding I-V curves of 10 µm distanced nanoprobes for an as-prepared (b) and heat-treated (c) IONC based mesocrystal are provided. Scale bar in inset (a) is 0.5 nm^{-1}.

Supporting Information 6: Binary Mesocrystals from Platinum and Iron Oxide Nanocubes

Two variants of binary mesocrystals have been synthesized according to a procedure, which we reported on in detail in literature.[14b] Both mesocrystals batches contain a combination of IONCs and PtNCs although in opposing amounts. **Figure S9a** shows FE-SEM images of a IONC based binary mesocrystal with incorporated small amounts of PtNC which can be seen in the high-resolution image (**Figure S9b**) due to the higher contrast of platinum already. A similar observation is made for an inverse superstructure (**Figure S9c**), where PtNCs form the host lattice while IONCs incorporate into it as shown in **Figure S9d**. This conclusion is

backed up by EDX point analysis of the binary mesocrystals' center (**Figure S9e**) as well as in depth investigations which we have presented in a preceding research article.[14b]

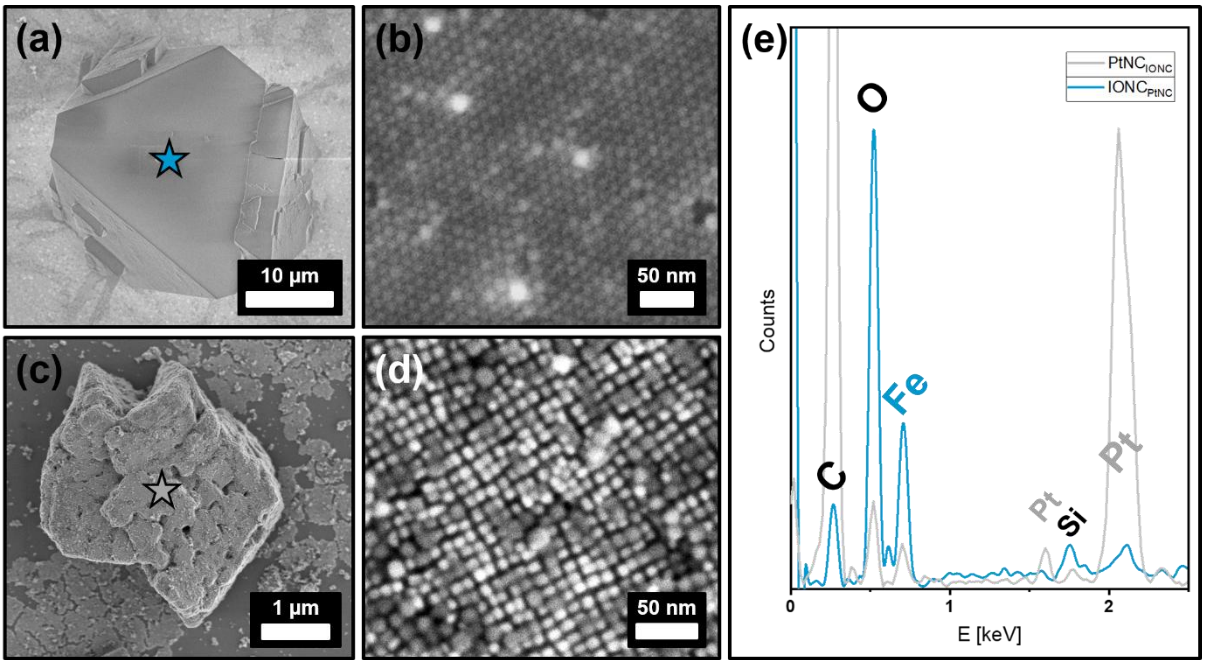

Figure S9. FE-SEM images of binary mesocrystals from IONCs and PtNCs. (a) shows an IONC host lattice containing small amounts of PtNCs as indicated by the bright spots in the high-resolution image of the binary mesocrystal surface in (b). Analogous, a PtNC host lattice binary mesocrystal with an incorporation of IONC is displayed in (c) and (d). This observation is backed up by point EDX analysis (e) at the center of both specimens marked with a star in (a) and (b).
